# Reading comprehension in children with Down syndrome

**DOI:** 10.1007/s11145-015-9578-8

**Published:** 2015-08-13

**Authors:** Glynis Laws, Heather Brown, Elizabeth Main

**Affiliations:** School of Experimental Psychology, 12a Priory Road, Bristol, BS8 1TU UK

**Keywords:** Down syndrome, Reading comprehension, Listening comprehension, Phonological awareness, Vocabulary knowledge

## Abstract

Two studies aimed to investigate the reading comprehension abilities of 14 readers with Down syndrome aged 6 years 8 months to 13 years relative to those of typically developing children matched on word reading ability, and to investigate how these abilities were associated with reading accuracy, listening comprehension, phonological awareness and vocabulary knowledge. Study 1 confirmed significantly poorer passage-reading comprehension than the typically developing group. In an experimental task, readers with Down syndrome understood fewer written sentences than the typical group and, contrary to prediction, received no advantage from printed sentences compared to spoken sentences, despite the lower memory load. Reading comprehension was associated with listening comprehension, word reading and phonological awareness in DS. Vocabulary knowledge was also associated with reading comprehension, mediated by word reading and nonverbal cognitive abilities. Study 2 investigated the longitudinal relationships between reading and language measures in the readers with DS over around 22 months. Time 1 listening comprehension and phonological awareness predicted Time 2 reading comprehension but there was no evidence that reading or reading comprehension predicted Time 2 language scores or phonological awareness, and no evidence that readers had acquired greater depth of vocabulary.

## Introduction

Down syndrome (DS) is the most common biological cause of intellectual disability, affecting 1.08 in 1000 live births in the UK (Morris & Alberman, 2009). Despite well-documented deficits in oral language (e.g., Abbeduto, Warren, & Conners, 2007), many individuals with DS learn to read (for reviews see: Kay-Raining Bird & Chapman, 2011; Laws, 2010; Næss, Melby-Lervåg, Hulme, & Lyster, 2012). Word recognition skills are usually more advanced than reading comprehension but few studies have investigated this discrepancy (see Ricketts, 2011). However, there has been progress in understanding the processes involved in reading comprehension in typical development (Oakhill & Cain, 2007; Perfetti, Landi, & Oakhill, 2005), including the causes of difficulties for *poor comprehenders*, that is children who have poor reading comprehension despite accurate word recognition (Cain & Oakhill, 2007; Nation, 2005a, b). The present studies draw on this research to investigate reading comprehension in children with DS.

In the simple view of reading (Gough & Tumner, 1986; Hoover & Gough, 1990) successful comprehension depends on a combination of printed word recognition and listening comprehension abilities. Since these abilities involve complex cognitive, linguistic and meta-linguistic skills and the interactions among them there are many ways in which reading comprehension is vulnerable to breakdown (Cain & Oakhill, 2006, 2007; Nation, 2005b). The convergent skills model of reading development (Vellutino, Tumner, Jaccard, & Chen, 2007) describes differences in the relative contributions of word identification and language comprehension to reading comprehension at different stages of reading development. For beginning readers, word identification skills and phonological abilities are of primary importance. Once word recognition skills are adequate, listening comprehension, encompassing semantic and syntactic abilities, assumes more importance.

Although most children with DS attending UK mainstream schools learn to identify words, they find comprehension tests difficult, particularly when verbal responses are required (Byrne, MacDonald, & Buckley, Byrne et al. 2002). Even good readers show marked discrepancy between standards of word recognition and reading comprehension (Boudreau, 2002; Byrne, et al., 2002; Cardoso-Martins, Peterson, Olson, & Pennington, 2009; Kay-Raining Bird, Cleave, & McConnell, 2000; Laws & Gunn, 2002; Groen, Laws, Nation, & Bishop, 2006; Roch & Levorato, 2009). Longitudinal studies suggest that these aspects of reading develop independently in DS (Byrne et al., 2002; Laws & Gunn, 2002; Roch, Florit, & Levorato, 2011), with slow progress in comprehension continuing into maturity (Carr, 1995; Fowler, Doherty, & Boynton, 1995; Moni & Jobling, 2001; Nash & Heath, 2011; Verucci, Menghini, & Vicari, 2006). This pattern of performance, and the fact that oral language abilities of readers with DS are usually below the developmental level predicted by word recognition skills (Boudreau, 2002; Fidler, Most, & Guiberson, 2005; Groen et al., 2006), suggests that listening comprehension presents the main barrier to reading with understanding.

Features of language impairment in DS include deficits in phonology (e.g., Stoel-Gammon, 1997) and in depth of vocabulary knowledge (Laws et al., 2014). It is worth considering whether such deficits affect reading comprehension beyond the roles specified for phonology and semantics in theories of word recognition (e.g., Plaut, McClelland, Seidenberg, & Patterson, 1996). Reading profiles of individuals with DS have been likened to those of poor comprehenders (Groen et al., 2006; Nash & Heath, 2011; Roch & Levorato, 2009). Nash and Heath (2011) compared the profiles of individuals with DS aged 11–19 years to those of younger typically developing children with reading comprehension at least 12 months below single word reading. Most individuals with DS could also be classified as poor comprehenders with delays of between 14 and 56 months between reading accuracy and comprehension ages. The groups had comparable receptive vocabulary but the poor comprehenders had significantly better decoding abilities consistent with other reports that poor comprehenders are not normally phonologically delayed (Stothard & Hulme, 1995). In contrast, individuals with DS have delayed or deficient phonological development, manifested in speech production difficulties (Dodd & Thompson, 2001; Stoel-Gammon, 1997), limited phonological awareness (PA; Lemons & Fuchs, 2010), and poor phonological memory (Cairns & Jarrold, 2005). Phonological memory is typically assessed by nonword repetition tasks that require individuals to hear and repeat unfamiliar strings of speech sounds. In DS, nonword repetition is associated with PA (Laws & Gunn, 2002) and with oral language development (Cairns & Jarrold, 2005; Laws, 2004; Miolo, Chapman, & Sindberg, 2005). Nonword repetition was closely related to sentence recall and mean length of utterance, even when variations in chronological age (CA), nonverbal cognitive abilities and real word repetition were statistically controlled (Laws, 2004). In the same individuals, nonword repetition and PA predicted reading comprehension 5 years later (Laws & Gunn, 2002). Taken together, these results suggest that the phonological deficit impacts on reading comprehension, mediated by verbal memory and/or oral language abilities.

Given phonological impairments, readers with DS may rely more on visual processing to link printed words directly to meaning (Fidler et al., 2005; Roch & Jarrold, 2008). Receptive vocabulary is usually a relative strength (e.g., Chapman, Schwartz, & Kay-Raining Bird, 1991) and predicts single word reading in DS (Fowler et al., 1995; Hulme, Goetz, Brigstock, Nash, Lervåg, & Snowling, 2012; Laws & Gunn, 2002). However, the relatively superficial understanding required to match words to pictures in receptive vocabulary tests may provide insufficient support for proficient reading comprehension. Such tests estimate *vocabulary breadth*, the number of phonological entries in the lexicon with associated meanings. The extent to which elaborated semantic knowledge is associated with these meanings is an indication of v*ocabulary depth* (Funnell, Hughes, & Woodcock, 2006; Ouellette, 2006). In typical development, vocabulary depth, revealed by children’s word definitions or attempts to describe how two things are alike, is linked to reading comprehension (Ouellette, 2006). Despite relative strength in receptive vocabulary, responses to a picture-based semantic association task revealed a significant deficit in vocabulary depth for children with DS relative to vocabulary breadth, and compared to typically developing children and children with specific language impairment matched on receptive vocabulary (Laws et al., 2014). The present study investigated whether this deficit is associated with reading comprehension.

Listening comprehension depends not only on understanding word meanings but also on deriving meaning from morpho-syntactic structure, an established difficulty for individuals with DS (e.g., Chapman et al., 1991; Laws & Bishop, 2003). Research to apply the simple view of reading to Italian adolescent readers with DS found listening comprehension was poorer than for typically developing children matched on reading comprehension, and more strongly correlated with reading comprehension in the group with DS (Roch & Levorato, 2009). Follow-up study of 10 individuals aged 11–20 years confirmed that listening comprehension rather than word recognition predicted reading comprehension (Roch et al., 2011). In contrast, earlier longitudinal studies that had assessed listening comprehension with separate tests of vocabulary and grammar understanding related both functions to word recognition rather than to reading comprehension (Byrne et al., 2002, Laws & Gunn, 2002). These results possibly reflected the importance of oral language skills for emerging printed word recognition since these studies included some limited and non-readers whereas Roch and colleagues studied readers with DS. Also, written Italian offers a more transparent orthography than English, which could influence the balance of contributions to reading comprehension from word recognition and listening comprehension. For Roch and Levorato’s (2009) ‘fast and accurate’ readers, listening comprehension could have more influence on reading comprehension. The present studies should extend our understanding of reading comprehension in DS by investigating English-speaking, younger readers.

## Outline of present studies

Participants were drawn from a longitudinal study of language and early reading development of children with DS attending mainstream primary schools at the start of the study. Towards the end of the study, around half the children had acquired sufficient reading skills to attempt reading comprehension assessments. In Study 1, these readers completed tests of reading comprehension along with single word reading, PA, receptive vocabulary, vocabulary knowledge, listening comprehension, and sentence recall. Results were compared to those of typically developing children matched on single word reading. Previous research predicts a deficit in reading comprehension for children with DS relative to the performance of the reading-matched group.

Roch and Levorato’s (2009) study, described above, compared listening and reading comprehension of paragraphs of text. However, to take account of the younger CAs and less mature reading and language abilities of our participants with DS, Study 1 compared comprehension of spoken and printed single sentences. Understanding of spoken sentences was assessed using a standardized test. A parallel version of the test was devised, consisting of written sentences of comparable linguistic difficulty, with picture-pointing responses required for both tasks. Given the deficits in short term memory associated with DS (Jarrold, Purser, & Brock, 2006), individuals may understand better the permanent, visual representations of written sentences than the transient representations offered by spoken sentences, which place more demands on memory (Buckley, 1993). The study also investigated associations between reading comprehension and the other measures.

Study 2 investigated the longitudinal relationships between the measures described in Study 1 with reading and language measures gathered around 2 years earlier. Research showing reciprocal relationships between reading and oral language in typical development (e.g., Stanovich, 1986), and anecdotal reports that some young children with DS learn new words from flashcards, has contributed to opinion that teaching reading will improve the oral language of children with DS (Alton, 2006; Buckley, 2000; Early Support, 2005; Oelwein, 1995). Associations between reading and language measures could be attributed either to oral language providing a foundation for literacy or to a beneficial effect of reading on oral language development. Children who understand what they read could be inclined to read more and increased exposure to language structure could benefit listening comprehension and PA. Similarly, greater vocabulary knowledge should support children’s reading comprehension but, equally, reading fiction and non-fiction with understanding should extend children’s knowledge. Understanding the nature of these relationships is important for informing the design of interventions.

Roch et al. (2011) found no evidence that reading benefited the listening comprehension of adolescents with DS; reading comprehension improved slightly over 1 year but listening comprehension did not change. However, as adolescents, Roch et al.’s participants could have reached a plateau in terms of morpho-syntactic development (Fowler, 1990, 1995; Laws & Gunn, 2004), limiting the potential for oral language benefits from reading. There could be more scope to benefit at the younger CAs of our participants. As a further investigation of the influence of reading on oral language, Study 2 compared the language scores and progress of readers with DS included in Study 1 with those of children who had been taking part in the same longitudinal study but whose progress had been too limited to attempt the reading comprehension assessments.

## Study 1: Reading and listening comprehension in children with down syndrome and typically developing children

### Methods

#### Participants

##### Children with DS

14 children (3 boys), aged 6 years 10 months to 13 years, with reading skills adequate to attempt the reading comprehension assessments, were selected from a group taking part in a longitudinal study. All the children attended mainstream primary schools at the start of the study and were being taught reading as part of the regular curriculum. By the time of this reading comprehension study, one child had moved to a mainstream secondary school and three children had moved to special schools. Parents confirmed that children spoke English as their first language and had no additional diagnoses of autism or other developmental disorders. One child had a history of hearing difficulties.

##### Typically developing children

Children with no learning or language difficulties were recruited to a comparison group matched on single word reading to the group with DS. Class teachers in three schools identified typical children thought to be reading as expected for CA. These were assessed using Word Reading from the British Ability Scales, second edition (BAS II; Elliott, 1996) and the British Picture Vocabulary Scale, version 2 (BPVS II; Dunn, Dunn, Whetton, & Burley, 1997). Those with age-appropriate reading and receptive vocabulary were invited to take part in the study and received the remaining assessments (see below). Four children with nonverbal IQs < 80 and/or standard scores of 3 on the Sentence Structure and Sentence Recall subtests from the Clinical Evaluation of Language Fundamentals, third edition UK (CELF-3UK; Semel, Wiig, & Secord, 2000) were excluded at this stage on the grounds that their reading profiles could also be atypical. The final comparison group included 23 children (12 boys). Parents confirmed that children spoke English as their first language and had no hearing loss or diagnoses of developmental disorders.

#### Assessment and measures

##### Nonverbal ability

Children with DS completed the Leiter International Performance Scale—Revised (Leiter-R; Roid & Miller, 1997). Following instructions in the manual, a brief nonverbal IQ was calculated and scores from four subtests (figure ground; form completion; sequential order; and repeated patterns) provided an estimate of nonverbal mental age (MA).

Typically developing children completed the Wechsler Abbreviated Scale of Intelligence (Wechsler, 1999) to screen for low IQ. Scores for the block design and matrix reasoning subtests provided a measure of nonverbal IQ. Age equivalent scores for the two subtests were averaged to estimate nonverbal MA.

##### Vocabulary

Receptive vocabulary (vocabulary breadth) was assessed by the BPVS II (Dunn et al., 1997). The examiner speaks a word and the child must choose the corresponding picture from a display of four. All children were tested from the beginning of the test rather than from starting points for CA indicated in the test instructions.

Vocabulary knowledge (vocabulary depth) was assessed using a 35-item picture-based semantic association test (Laws et al., 2014), modelled on the Camel and Cactus Test for adults with semantic dementia (Bozeat, Ralph, Patterson, Garrard, & Hodges, 2000). The test was programmed using E-Prime and presented on a 15-inch Elo USB Touchscreen controlled by a laptop computer. Target pictures (e.g., *baby*) appeared at the top of the screen and four pictured alternatives (in this example: *pram*^1^ (the correct response), *wheelbarrow*, *go*-*kart*, *bicycle*) appeared simultaneously in a row beneath. For all items, response choices were semantically related but only the correct response was related to the target. For the majority of items, the semantic relationship between target and response depended on function (e.g., *baby* and *pram*). Other relationships depended on analogy (e.g., *tortoise* and *snail*), shared category membership (e.g., *eagle* and *owl*), or general knowledge (e.g., *arrow* and *Robin Hood*). Children were directed to view the response pictures and to touch or point to the one that went with the target picture. Items were presented in one of four fixed orders; the position of the correct response picture was varied across items. All participants demonstrated understanding of the task by responding correctly to two easy practice items before proceeding.

##### Phonological awareness

Children completed six PA tasks, all requiring picture-pointing responses. Each task began with two training items with feedback followed by nine test items with no feedback.

1. Rime segmentation and matching was assessed following a procedure described by Bird, Bishop, and Freeman (1995). Children were shown a picture of a monster and told, for example, “This is Dan. Dan likes words that sound like his name”. An array of four pictures was presented and the experimenter asked: “Would Dan like a spoon, a ring, a pan or a key?”, naming each picture as she pointed to it. Children were instructed to point to the picture that they thought Dan would choose. The position of the correct response picture was varied across items.
2. Onset segmentation and matching was assessed using the same procedure except that children were asked to point to the picture that started with the same sound as the monster’s name.
3. Onset matching followed a similar procedure except that the task was modified to remove the requirement to segment the onset sound. Instead, the examiner provided the onset sound. After naming the four pictures and repeating the onset sound, she asked the child to point to the picture that started with the same sound.
4. Word blending investigated whether children could put two words together to make a third word (e.g., *butter* and *fly*, with *butterfly* as the target response). Two finger puppets, one on each of the examiner’s hands, ‘spoke’ the elements of the target word. Children were asked to listen and then point to the picture representing the target word.
5. Onset phoneme and rime blending investigated whether children could blend word onsets with rimes (e.g., *f,* -*ish*). The first puppet ‘spoke’ the onset sound and the second puppet ‘spoke’ the rime sound before children were asked to point to the picture showing the resulting word.
6. Consonant deletion required children to listen to a word and then choose the picture that showed the word that would remain if a sound was removed (e.g., *swing* becomes *wing* if /s/ is removed). All items involved the deletion of an onset phoneme (either /s/, /b/, or /t/).

##### Sentence recall

The Sentence Recall subtest from the CELF-3UK (Semel et al., 2000) assessed memory for sentences by asking children to repeat sentences spoken by the examiner. This is a complex task, sensitive to children’s grammatical weaknesses, speech and expressive language difficulties, as well as short-term memory limitations. Sentences increase in length and grammatical complexity as the test progresses. Scores for each sentence range from 3 for errorless recall to 0 if there are 4 errors or more. The test is discontinued after three consecutive zero scores.

##### Reading

Single word reading was assessed using BAS II Word Reading (Elliott, 1996). Text reading accuracy and reading comprehension were assessed using the Neale Analysis of Reading Ability-Revised (NARA II; Neale, 1997). Children read graded passages aloud, corrected by the examiner where necessary. Following the child’s reading, the examiner asks questions to probe understanding of the main narrative, the sequence of events and other details. Children are required to respond verbally. All participants started at the beginning of the test.

##### Comparison of listening and reading comprehension

Listening comprehension depends on children’s ability to make use of morphology and grammar to extract meaning from sentences, and also requires that they understand the vocabulary in those sentences. It was assessed using the Sentence Structure subtest from CELF-3UK (Semel et al., 2000). For each of 20 items, the examiner reads a short sentence and the child must point to the picture that illustrates it, choosing from an array of four alternatives. All items were administered.

Reading comprehension was assessed using a parallel version of the listening comprehension test, consisting of 20 printed sentences each written to correspond to one of the alternative response pictures in the arrays offered for the listening version. Offering different sentences to assess listening and reading comprehension avoided potential practice effects. In most cases, the printed sentences involved a semantic change to the spoken item. For example, one practice item for the listening comprehension test was: The boy has a ball. The reading comprehension version was: The girl has a ball. The same sentence structures were tested in both versions, if not always in the corresponding items. For example, item 1 in the listening version was: The boy is not climbing. The corresponding printed item was: The boy is climbing the frame. However, for balance, a printed sentence included a negative elsewhere in the test. Each sentence was printed on a card and children read it aloud. The sentence remained in view while a response picture was selected. Half the children completed the listening comprehension task first followed by reading comprehension and the others completed the tasks in the opposite order. No feedback was offered.

### Procedures

The Faculty of Science Research Ethics Committee (REC) at the University of Bristol and Gloucestershire National Health Service REC approved the research. All parents received information about the study and returned signed consent forms. Children were assessed in a quiet room in school over two to three sessions.

## Results

One child in each group did not manage the NARA II but did read and respond to the printed sentence comprehension task so their results were included in the study. Four typically developing children were not available for PA testing. Table 1 shows the CA, nonverbal IQ and MA and test scores for both groups. Raw scores for all reading and language tests were used in the analyses. The scores of boys and girls did not differ statistically significantly in either group.

**Table 1:** Mean scores for the group with DS and the typically developing group in Study 1

Variable (maximum score)	TD (n = 23)	DS (n = 14)	*t*	*p*	95 % CI	*d*
	*M (SD)*	*M (SD)*				
CA^a^	7.1 (0.8)	10.1 (1.8)	6.39 ^b^	.0001	2.0, 4.0	2.35
Nonverbal IQ	98.74 (9.49)	45.36 (8.19)	−17.44	.0001	−59.6, −47.2	5.15
Nonverbal MA^a^	6.10 (1.03)	4.6 (0.7)	−6.277	.0001	−3.01, −1.07	2.31
BPVS II rs (168)	78.65 (9.21)	54.71 (11.19)	−7.01	.0001	−30.8, −17.0	2.34
Vocabulary depth (35)	28.52 (2.94)	15.14 (6.87)	−6.91^b^	.0001	−17.5, −9.27	2.53
PA (54)	48.84 (2.50)^c^	33.64 (7.23)	−7.54^b^	.0001	−19.49, −10.91	2.81
CELF-3UK sentence recall rs (78)	29.96 (10.21)	3.79 (4.63)	−10.63	.0001	−31.2, −21.2	3.30
CELF-3UK sentence structure rs (20)	16.43 (2.33)	8.14 (3.51)	−8.66	.0001	10.25, −6.34	2.79
Printed sentence task (20)	14.78 (3.69)	8.07 (4.25)	−5.065	.0001	−9.40, −4.02	
BAS II word reading rs (90)	37.57 (18.70)	38.07 (15.24)	.085	.932	−11.51, 12.55	0.03
NARA II accuracy rs	28.14 (15.02)^d^	29.00 (12.99)^e^	.172	.864	−9.33, 11.05	0.06
NARA II comprehension rs	11.05 (6.37)^d^	3.46 (2.90)^e^	−4.08^b^	.0001	−11.41, −3.76	1.42

TD = typically developing; DS = Down syndrome; CI = confidence interval; *d* = *Cohen’s d;* CA = chronological age; MA = mental age; Nonverbal MA and Nonverbal IQ (TD: derived from Wechsler Abbreviated Scale of Intelligence; DS: derived from Leiter International Performance Scale-Revised); BPVS II = British picture vocabulary scales, 2nd edition; PA = phonological awareness; CELF-3UK = Clinical Evaluation of Language Fundamentals, third edition UK; BAS II = British ability scales, 2nd edition; NARA II = Neale Analysis of Reading Ability-Revised; rs = raw score

^a^ Years; months, ^b^ df adjusted for unequal variances, ^c^ n = 19, ^d^ n = 22, ^e^ n = 13

There were no statistically significant differences between the groups on BAS Word reading or NARA II Accuracy (*p*s = .93 and .86, respectively). Following Kover and Atwood (2013), effect sizes and variance ratios were inspected; effect sizes were close to 0 (Cohen’s *d* = .03 and .06, respectively), and variance ratios were .66 and .75 respectively, so group matching was deemed satisfactory. Despite comparable single word and passage reading accuracy, the mean NARA II reading comprehension score for the group with DS was significantly lower than for the typically developing group (*p* < .0001).

### Comparison of listening and reading comprehension

**Fig. 1:**
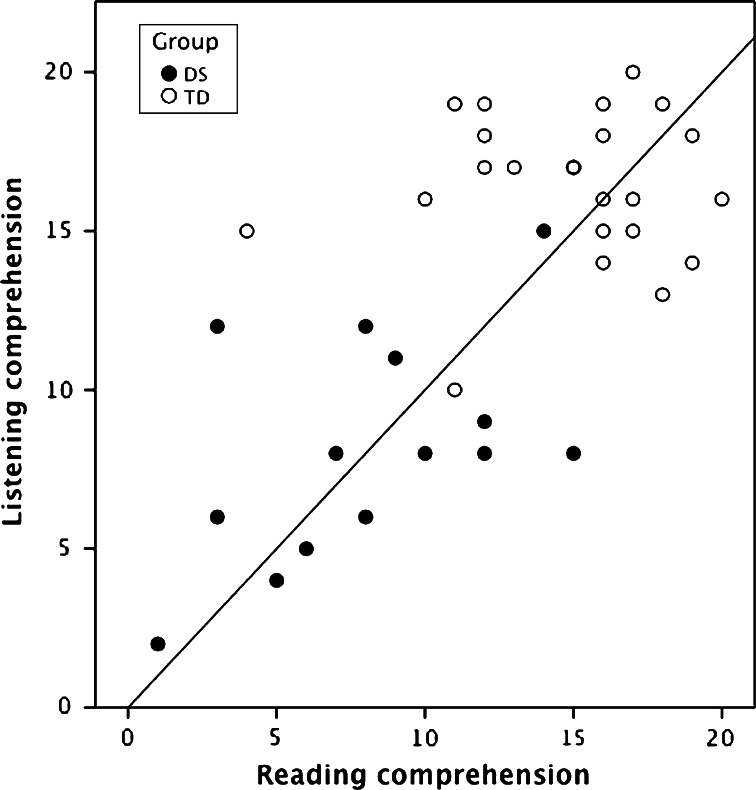
Scatterplot showing association between spoken and printed sentence comprehension scores for children with DS and typically developing children in Study 1

Figure 1 shows a scatterplot of scores obtained on the listening and reading sentence comprehension tasks by children in each group, illustrating the lower scores obtained by children with DS, and suggesting a closer relationship between the tasks in this group.

Mean listening and reading comprehension scores were higher for the typical group than for the group with DS (see Table 1). A mixed ANOVA to investigate group differences with sentence task as the repeated measure found a significant effect of group, *F* (1, 35) = 66.035, *p* < .0001, η_p_^2^ = .654. However, there was no significant effect of task, *F* (1, 35) = 1.533, *p* = .224, η_p_^2^ = .042, and no significant interaction between group and task, *F* (1, 35) = 1.290, *p* = .264, η_p_^2^ = .036. Children with DS performed at a lower level than typical children but, within groups, mean reading and listening comprehension scores did not differ. There was a statistically significant correlation between the tasks within the group with DS, *r* (14) = .510, *p* = .031, but no correlation within the typical group, *r* (23) = .070, *p* = .376.

### Associations between reading comprehension and other measures

Correlations and partial correlations were calculated to investigate whether the groups differed in the ways in which word reading and language measures were associated with reading comprehension, and to understand whether PA and depth of vocabulary knowledge were associated with reading comprehension in DS, independent of the contribution that both may make to word reading.

Across groups, passage and sentence reading comprehension scores were closely correlated, *r* (35) = .817, *p* = .0001, so a reading comprehension composite (RCC) was calculated as the sum of these scores (DS: *M* = 12.08; *SD* = 8.18; TD: *M* = 25.68; *SD* = 9.58; *t* (35) = −4.85, *p* < .0001). Similarly, single word and passage reading accuracy were closely correlated, *r* (35) = .901, *p* = .0001, so scores were summed to derive a reading composite (RC; DS: *M* = 65.00; *SD* = 29.47; TD: *M* = 64.48; *SD* = 32.9; *t* (35) = .049, *p* = .962). Correlations between the RCC, RC, and other measures were calculated within each group (see Table 2).

**Table 2:** Correlations between reading, reading comprehension and listening comprehension with other measures in both groups in Study 1

Measure	DS (n = 14)			TD (n = 23)		
	RCC	RC	LC	RCC	RC	LC
CA (months)	.40	.59*	.40	.60**	.55**	.12
MA (months)	.70**	.38	.74**	.04	−.04	.38
RCC	–	.65**	.56*	–	.90***	−.04
RC	.65**	–	.40	.90***	–	−.02
Listening comprehension	.56*	.40	–	−.04	−.02	–
Phonological awareness	.84 ***	.54*	.67**	.15	.07	−.16
Receptive vocabulary	.40	.32	.39	.38*	.36*	.05
Vocabulary depth	.64**	.64**	.46	.07	.02	.23
Sentence recall	.68 ***	.17	.72***	.36*	.33	−.05

RCC = reading comprehension composite, RC = reading composite, LC = listening comprehension, CA = chronological age, MA = mental age

*** *p* < .0001; ** *p* < .005; * *p* < .05

The pattern of correlations obtained for the group with DS differed from the pattern for the typical group. For typical children, CA was significantly correlated with the RC and RCC scores but, for children with DS, CA was associated with the RC but not with the RCC. Nonverbal MA did not correlate with the RC or the RCC in the typical group but there was a large and statistically significant correlation with the RCC in the group with DS. The RC was correlated with the RCC score in both groups; this was not simply because NARA II results contributed to both composite scores because BAS II single word reading was also significantly correlated with the RCC, *r* (23) = .856, *p* < .0001. There were moderate correlations between receptive vocabulary and sentence recall with the RCC in the typical group. There was no correlation between PA and the RC in the typical group due to the limited variation in PA scores (see Table 1). There was more variation in PA scores in the group with DS and PA, vocabulary depth and sentence memory were all associated with reading comprehension.

### Phonological awareness and reading comprehension in DS

Mean PA scores for the group with DS were statistically significantly lower than for the typical group (see Table 1) and were significantly correlated with the RCC, with the RC, and with listening comprehension (see Table 2). To investigate the possibility that the association between PA and reading comprehension was mediated by word reading, a partial correlation was calculated, controlling for RC scores, *r* (11) = .762, *p* = .002. This result suggests that the association between PA and reading comprehension was not attributable to the contribution PA makes to word reading. To investigate whether listening comprehension might mediate the relationship, listening comprehension scores were also controlled in a further partial correlation, *r* (10) = .697, *p* = .012. It was possible that all scores were constrained by children’s non-verbal MA. However, partial correlation controlling for Leiter MA in addition to reading and listening comprehension made little difference to the size of the correlation, *r* (9) = .672, *p* = .023.

### Vocabulary

Mean vocabulary breadth and depth were significantly lower for the group with DS (see Table 1). Although there were no significant correlations between vocabulary breadth and the RCC or RC, vocabulary depth was correlated with the RCC and with the RC (see Table 2). To investigate whether the association with the RCC was mediated by word recognition, a partial correlation was calculated controlling for RC scores, *r* (11) = .391, *p* = .187. Vocabulary depth and the RCC were both associated with nonverbal MA, so a further partial correlation was calculated controlling for nonverbal MA, *r* (10) = .188, *p* = .559.

### Sentence recall

Children with DS experienced considerable difficulties in recalling sentences (see Table 1) and there were significant correlations between sentence recall and the RCC and listening comprehension (see Table 2). There was also a significant correlation between sentence recall and PA, *r* (14) = .697, *p* = .006. Calculation of a partial correlation between sentence recall and the RCC, controlling for PA, produced no significant result, *r* (13) = .153, *p* = .544, suggesting that the association had been mediated by PA. This seemed to be the case because, when sentence recall was controlled, there remained a significant correlation between PA and the RCC, *r* (13) = .857, *p* < .0001.

## Discusssion

Reading comprehension of children with DS was significantly poorer than that of typical children matched on single word and text reading accuracy. Contrary to prediction, readers with DS found printed sentences no easier to understand than spoken sentences. As in other studies (Nash & Heath, 2011; Roch & Levorato, 2009), listening comprehension was more closely associated with reading comprehension in readers with DS than in the typical group. In Nash and Heath’s study this was thought due to their more advanced word reading because the groups had been matched on reading comprehension. However, in our study, the groups were reading-matched. Word reading was also associated with reading comprehension in our children with DS. As they were younger and had less mature reading skills than the adolescents in those earlier studies, perhaps more cognitive resources were devoted to decoding the words. Word reading was even more strongly correlated with reading comprehension in the typical group, and comparable to results described by Vellutino et al. (2007) for children at a similar stage of literacy acquisition.

There was marked association between PA and reading comprehension in the group with DS. Even when that part of the correlation mediated by word recognition was controlled, PA contributed 58 % to the variance in reading comprehension scores, with little change when listening comprehension and nonverbal MA were controlled. Vocabulary depth was also correlated with reading comprehension but this association appeared to be mediated by word reading and nonverbal MA. Finally, poor sentence recall was associated with reading comprehension, but not after controlling for PA. Potentially, this could be due to the requirement to access phonological representations in constructing the imitated sentences. Study 2 describes a further investigation of these relationships between language and reading comprehension.

## Study 2: Longitudinal relationships between listening comprehension, PA and vocabulary depth with reading comprehension in children with down syndrome

The reading comprehension measures described in Study 1 were gathered towards the end (Time 2) of a longitudinal study, on average 22 months after it began (Time 1). By investigating the relationships between Time 2 measures and reading and language measures from Time 1, we aimed to clarify the nature of the associations between reading comprehension and language measures described in Study 1. In particular, did reading comprehension proficiency build on oral language skills or did reading with understanding contribute to children’s oral language abilities?

Small sample size and the number of variables contributing to reading comprehension scores meant that requirements for multiple regression analyses were not met. An alternative approach to investigating longitudinal relationships is to calculate the partial correlation between each language measure at Time 1 and reading comprehension at Time 2, controlling for reading comprehension at Time 1. The reciprocal relationship is investigated by calculating the partial correlation between reading comprehension at Time 1 and each language measure at Time 2, controlling for the Time 1 language score. This approach was modified because different reading comprehension assessments had been administered at Times 1 and 2. At this early stage of reading, few children would have managed the NARA II assessment at Time 1 so scores were not available to use as the autoregressor in the partial correlations. However, scores obtained on a simpler reading comprehension test (see below) were significantly correlated with Time 2 RCC scores (*r* = .723, *p* = .002), so could account for the reading comprehension abilities that children had started out with. Similarly, scores on a language comprehension measure at Time 1 were significantly correlated with the Time 2 listening comprehension measure (*r* = .801, *p* < .0001), so took account of children’s listening comprehension abilities at the start of the study.

Partial correlations investigated the reciprocal relationships between reading comprehension and receptive vocabulary and PA assessments completed at Times 1 and 2. Since vocabulary depth had not been assessed at Time 1, it was not possible to investigate whether it predicted reading comprehension. However, it was possible to investigate whether reading with understanding helped children to elaborate their vocabulary knowledge. The vocabulary knowledge of the better readers was compared to that of the limited readers who had not completed the reading comprehension assessments. If reading contributed to children’s vocabulary knowledge, then the better readers should have achieved greater vocabulary knowledge by Time 2 than the limited readers.

## Methods

### Participants

Two groups of children with DS were included in Study 2: the 14 readers described in Study 1; and 14 children (7 boys), aged 6 years 8 months to 11 years 10 months, who had not taken part because their reading abilities were limited. There were trends for this group to be younger on average and of lower nonverbal MA (CA: *p* = .066; Nonverbal MA: *p* = .097). They also differed significantly in language comprehension at Time 1, *t* (26) = −3.544, *p* = .002, and in receptive vocabulary, *t* (26) = 3.337, *p* = .003. One limited reader had moved to a special school by Time 2. Whereas only 1 reader had hearing difficulties, 8 limited readers had hearing losses reported either by parents or audiologists. These losses contributed to poor reading progress (Laws, 2010).

### Assessment and measures

Some Time 2 (Study 1) measures had also been administered at Time 1: Leiter R (Roid & Miller, 1997), BPVS II (Dunn et al., 1997), BAS II Word Reading (Elliott, 1996), and the PA tasks. Other measures administered at Time 1 included:

#### Reading comprehension

Children were offered Reading: Understanding from the Kaufman Assessment Battery for Children (Kaufman & Kaufman, 1983), which involves no verbal questioning and requires no spoken responses. Instead, children mime what they read; for example, children read ‘Stand’ and respond by standing up.

#### Listening comprehension

Children completed the Reynell Developmental Language Scales (RDLS; Edwards et al., 1997). The RDLS provides a clinical assessment of expressive language and comprehension for children aged 18 months to 7 years. The comprehension scale scores were used as the Time 1 listening comprehension measure. The assessment covers 11 sections, beginning with understanding of single words. Subsequent sections increase in difficulty, following the usual sequence of language development; the last sections assess more complex grammar and inferencing ability.

## Results

Table 3 describes reading and language scores at Times 1 and 2 for readers included in Study 1 and those of the children with limited reading.

**Table 3:** Comparison of CA, nonverbal MA, and language scores of readers and limited readers with DS at Times 1 and 2

Variable (maximum score)	Readers (n = 14)		Limited readers (n = 14)	
	Time 1	Time 2	Time 1	Time 2
	*M (SD)*	*M (SD)*	*M (SD)*	*M (SD)*
CA^a^	8;5 (1;8)	10;1 (1;8)	6;11 (1;6)	9;0 (1;5)
Nonverbal MA^a^	4;2 (0;7)	4;7 (0;7)	3;7 (0;8)	4;1 (0;8)
BAS II Word reading rs (90)	23.64 (15.67)	38.07 (15.24)	1.79 (2.64)	7.54 (5.92)
K-ABC reading Understanding (24)	3.29 (3.07)	N/A	0	N/A
BPVS II rs (168)	45.43 (11.47)	54.71 (11.19)	29.29 (14.00)	34.64 (17.60)
Vocabulary depth (35)	N/A	15.14 (6.87)	N/A	13.57 (6.1)
Phonological Awareness (54)	29.00 (7.64)	33.64 (7.23)	16.85 (8.71)	18.77 (11.97)
CELF-3UK Sentence structure rs (20)	N/A	8.14 (3.51)	N/A	6.50 (2.82)
RDLS Comprehension (62)	46.07 (6.11)	N/A	32.14 (13.38)	N/A

CA = chronological age; MA = mental age; BAS II = British Ability Scales, 2nd edition; K-ABC = Kaufman Assessment Battery for Children; BPVSII = British Picture Vocabulary Scales, 2nd edition; Clinical Evaluation of Language Fundamentals, third edition UK; RDLS = Reynell Developmental Language Scales; N/A = not assessed

Investigations of the reciprocal relationships between reading comprehension and other measures are restricted to readers’ results since no child in the limited reading group achieved a reading comprehension score. The partial correlation between readers’ nonverbal MA at Time 1 and reading comprehension (the RCC) at Time 2, taking reading comprehension at Time 1 into account, was not statistically significant, *r* (11) = 396, *p* = .09. Time 1 single word reading was a significant predictor of word reading (RC) at Time 2, *r* (14) = .714, *p* = .002, and a significant predictor of Time 2 reading comprehension (RCC), *r* (14) = 574, *p* = .016, but not when Time 1 reading comprehension was controlled, *r* (11) = -.362, *p* = .112.

### Relationship between listening comprehension and reading comprehension

Given the associations between language and reading measures reported in Study 1, and previous research findings, one-tailed *p* values for correlations are reported.

**Fig. 2:**
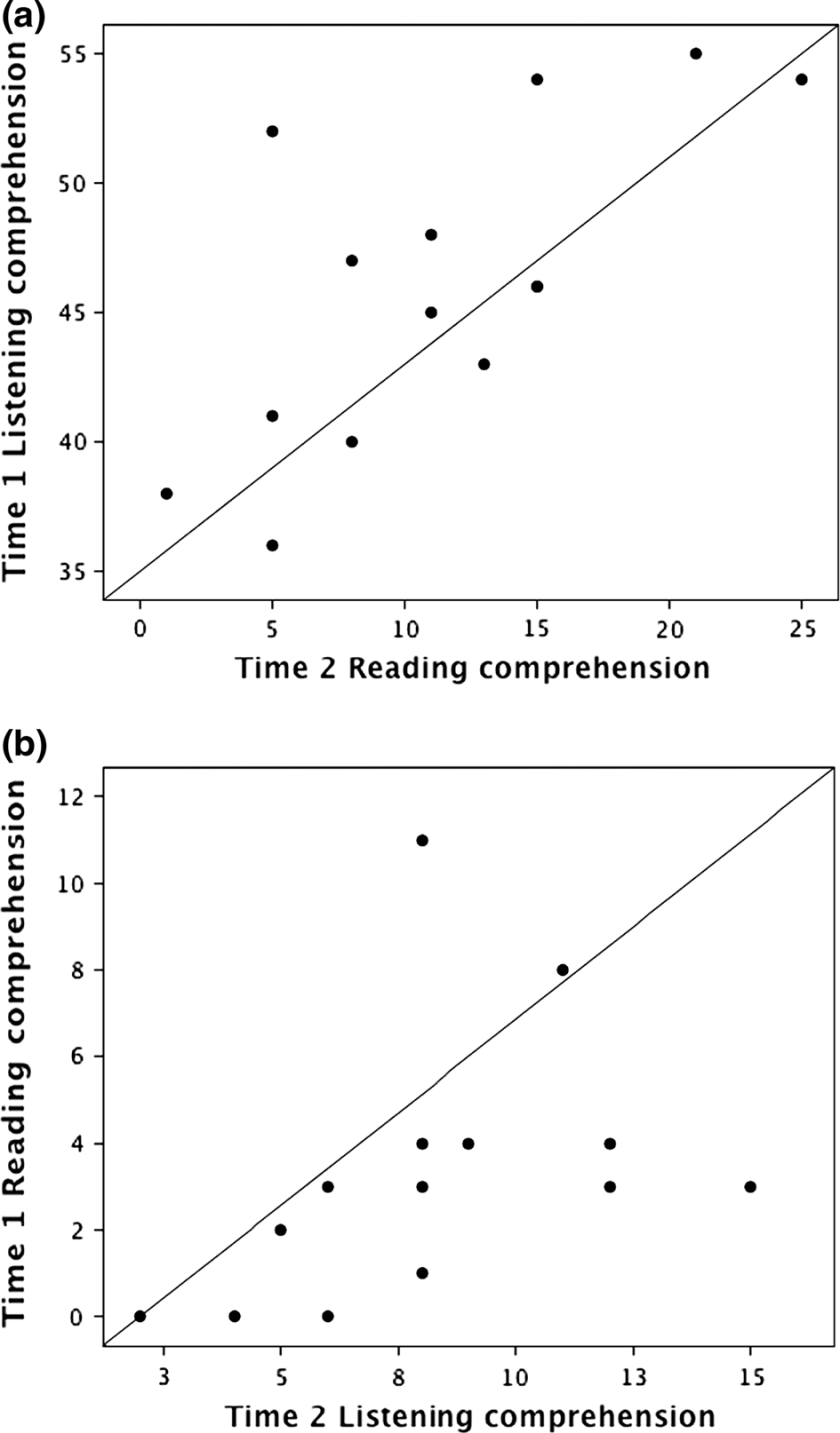
**a** Scatterplot showing relationship between Time 1 listening comprehension and Time 2 reading comprehension in Study 2. **b** Scatterplot showing relationship between Time 1 reading comprehension and Time 2 listening comprehension in Study 2

Figure 2a shows a scatterplot of Time 1 listening comprehension against Time 2 reading comprehension scores, which suggests a relationship between these measures. A partial correlation was calculated, controlling for Time 1 reading comprehension, *r* (11) = .416, *p* = .079. One older girl (aged 11 years 8 months) had had one of the best listening comprehension scores at Time 1 but developed unexpectedly poor reading comprehension. When her results were excluded from the analysis, there was a substantial correlation between these variables in the remaining group, *r* (11) = .856, *p* < .0001. The reciprocal relationship, illustrated in Fig. 2b, was investigated by calculating the partial correlation between Time 1 reading comprehension and Time 2 listening comprehension, controlling for Time 1 listening comprehension, *r* (11) = −.445, *p* = .064.

Reading comprehension scores at Time 1 were somewhat restricted but it was plausible that the greater variability in single word reading scores at Time 1 could be more revealing of an effect of reading on listening comprehension at Time 2. With this in mind, the partial correlation was recalculated substituting single word reading scores as the Time 1 measure, *r* (11) = −.429, *p* = .072. In sum, Time 1 listening comprehension predicted Time 2 reading comprehension scores but neither reading nor reading comprehension at Time 1 predicted listening comprehension at Time 2.

### Relationship between phonological awareness and reading comprehension

A partial correlation was calculated between Time 1 PA and reading comprehension at Time 2 (the RCC), controlling for Time 1 reading comprehension, *r* (11) = .537, *p* = .029. The reciprocal relationship was investigated by calculating the partial correlation between Time 1 reading comprehension and PA at Time 2, controlling for Time 1 PA, *r* (11) = .315, *p* = .294. Following the reasoning above, this partial correlation was recalculated substituting Time 1 single word reading scores, *r* (11) = .173, *p* = .573. For completeness, a partial correlation was calculated between PA at Time 1 and single word reading at Time 2, controlling for single word reading at Time 1, *r* (11) = .669, *p* = .012. In sum, PA at Time 1 predicted reading and reading comprehension at Time 2 but neither reading nor reading comprehension at Time 1 were significant predictors of PA at Time 2.

As a further investigation, the PA scores of the better readers were compared to those of the limited readers (see Table 3). The readers had greater PA at Times 1 and 2. If increased exposure to speech sounds through reading helps develop PA, they should have made relatively more progress in PA over the course of the study. A mixed ANOVA investigated group differences, with PA at Times 1 and 2 as the repeated measure. Since the readers differed from the limited readers not only in reading but also in receptive vocabulary with a trend towards lower nonverbal MAs, BPVS II and Leiter MA scores were treated as co-variates. Group membership had a significant effect on PA scores, *F* (1, 24) = 6.298, *p* = .02, η_p_^2^ = .215, as did Leiter MA, *F* (1, 24) = 7.077, *p* = .041, η_p_^2^ = .235 and BPVS II scores, *F* (1, 24) = 5.23, *p* = .032, η_p_^2^ = .185. However, the repeated measure showed no significant effect, *F* (1, 24) = .001, *p* = .982, η_p_^2^ = .000. There were no significant interactions including, critically, no significant interaction between group membership and the repeated measure, *F* (1, 24) = .011, *p* = .917, η_p_^2^ = .000. In sum, although the better readers had greater PA than limited readers, and this contributed to reading comprehension, there was no evidence that reading experience produced greater increases in PA over the course of this study.

### Vocabulary and reading comprehension

Although there was no concurrent correlation between receptive vocabulary scores and reading comprehension in Study 1, a partial correlation between Time 1 receptive vocabulary and the RCC at Time 2 was calculated, controlling for Time 1 reading comprehension, *r* (11) = .382, *p* = .197. The reciprocal relationship was investigated by partial correlation between Time 1 reading comprehension and Time 2 receptive vocabulary scores, controlling for Time 1 receptive vocabulary, *r* (11) = −.237, *p* = .435. Although readers had better vocabulary than limited readers (see Table 3), there was no suggestion that reading predicted receptive vocabulary progress. Despite this, it was still plausible that reading with understanding could deepen children’s vocabulary knowledge. An ANCOVA investigated the difference between groups’ mean scores on the Time 2 vocabulary depth measure, treating Leiter MA as a covariate. Although Leiter MA had a significant effect on vocabulary depth, *F* (1, 25) = 7.039, *p* = .014, η_p_^2^ = .22, there was no effect of group, *F* (1, 25) = .031, *p* = .863, η_p_^2^ = .001. In sum, there was no evidence that the better readers had greater depth of vocabulary knowledge than the limited readers.

## Discussion

Time 1 listening comprehension was an indicator of reading comprehension at Time 2. Time 1 PA was also a significant predictor of Time 2 reading comprehension but the reduction in the size of the partial correlation when word reading scores were controlled suggested that the relationship was, in part, mediated by word recognition skills.

Neither reading comprehension nor single word reading at Time 1 predicted listening comprehension or PA at Time 2. As a further investigation, PA scores obtained by the readers at Time 2 were compared to those of the limited readers. Although the readers had superior PA at Times 1 and 2, and this was related to their better receptive vocabulary, there was no evidence of greater growth in PA for this group over the course of the study. This was not due to readers reaching a test ceiling by Time 2 since scope remained for all of them to increase scores. Taken together, these analyses suggest that PA is important to emerging literacy in children with DS but that reading itself does not advance their PA. Readers did not differ from limited readers on the vocabulary depth measure at Time 2, suggesting that reading was not contributing to the elaboration of readers’ vocabulary knowledge.

## Summary and concluding discussion

Since many children with DS progress in reading words it is important to understand why this ability is not matched by reading comprehension so that, potentially, children could be helped to get more from their reading. Study 1 showed that some readers with DS clearly understand some of what they read when comprehension is tested without the requirement for verbal responses. Despite low scores on the standardised passage reading comprehension test, 10/14 children scored above chance when comprehension of printed sentences was assessed using picture-pointing responses.

Consistent with other reports of a closer association between listening and reading comprehension in individuals with DS (Nash & Heath, 2011; Roch et al., 2011), Study 1 produced a stronger relationship between spoken and printed sentence comprehension in the group with DS than in the typical group. Unlike Roch and colleagues’ findings, word reading was also associated with reading comprehension in the children with DS, perhaps because they were younger, less advanced readers. However, it accounted for a larger proportion (81 %) of variation in the reading comprehension scores of the typical group, which perhaps left less room for listening comprehension to contribute to variance. This correlation is comparable to that reported by Vellutino et al. (2007) for typical children at the same stage of reading. The results are also compatible with parallel progress in word reading and reading comprehension in typical development in contrast to separate pathways of development evident for this group with DS and in previous research studies.

The importance of listening comprehension to reading with understanding by children with DS was confirmed in Study 2. Listening comprehension, based on a measure tapping semantic and syntactic abilities, predicted reading comprehension over about 2 years in the majority of the group. However, the case of an 11-year-old girl with poor reading comprehension despite good listening comprehension and reading accuracy should also be noted. The heterogeneous nature of abilities in DS means listening comprehension may not always be a reliable indicator of a child’s ability to read with understanding.

The spoken and printed sentences in Study 1 were designed to offer comparable linguistic difficulty and so were perhaps likely to elicit comparable scores, but the same cannot be argued for the RDLS administered at Time 1. This test differentiated readers from limited readers and also predicted readers’ comprehension scores by Time 2. Among readers, the main variation in RDLS scores arose from performance on subtests of complex grammar and inferencing. Performance was poorest across 8 items testing inferencing (median score = 2.5; range 0–5). Interestingly, on the Time 2 printed sentence comprehension task, not one reader succeeded in matching “The girl is wishing she had worn her new coat” to a picture of a girl in the rain with no coat, supporting suggestions that poor inferencing limits the comprehension of readers with DS (Groen et al., 2006; Nash & Heath, 2011). Further research to locate the source of inference difficulties in DS would be of value. In poor comprehenders, this does not necessarily lie with syntactic processing difficulties or limited general knowledge (Cain & Oakhill, 2007) but both of these factors can influence reading comprehension in DS.

The strong association between PA and reading comprehension in Study 1 was not accounted for by the contribution of PA to word reading or variation in children’s listening comprehension or nonverbal MA. Study 2 confirmed PA as a predictor of reading comprehension and word reading, with the contribution from PA to word reading accounting for only part of the variation in reading comprehension scores. In typically developing young readers, PA contributes directly to reading comprehension, independent of working memory (Leather & Henry, 1994). Although no working memory tests were applied in the present study, controlling for sentence memory did not eliminate the correlation between PA and reading comprehension. Further research is necessary to understand the role that PA plays in reading comprehension in DS.

Contrary to prediction, children with DS did not find the printed sentences easier to understand than spoken sentences. One exception was an older boy with a history of hearing loss who understood 15/20 printed sentences compared to 8/20 spoken sentences. Print may have compensated for his hearing difficulties but, in general, reading did not overcome them because other children with hearing losses had made little progress in reading. The fact that children found it no easier to understand printed sentences is at odds with Buckley’s (1993) report of some teenagers with DS who learned to understand grammatical structures when supported by written sentences. Buckley (1993) hypothesized that print overcame the auditory short-term memory deficits associated with the syndrome. However, while this may have helped after 1 year of training that had provided repeated practice of the sentence structures, printed sentences did not support comprehension in the assessment situation offered in Study 1. Mengoni, Nash, and Hulme (2013) also drew attention to the disparity between children’s learning in a training experiment and long-term gains in vocabulary. They found some children with DS gained a small advantage in learning the names of novel objects when provided with orthographic support. Learning was comparable to that achieved by a reading-matched group of typical children but Mengoni et al. (2013) point out that if children with DS actually acquired vocabulary at a comparable rate then their vocabulary should grow at similar rates, which it does not. It is worth noting that the post-test in their experiment was applied 10–15 min after the training with no follow-up assessment and also, since the printed words remained in view, there was no evidence that words had been added to children’s receptive vocabularies even in the short term.

Study 2 found that neither Time 1 single word reading nor reading comprehension predicted listening comprehension or PA at Time 2. Children with DS would be expected to make less language progress over this period than typically developing children but it is noteworthy that readers made no more progress on these measures than limited readers. Similar results were obtained comparing progress on receptive vocabulary, verbal short-term memory and speech production tests for good and poor readers (Laws, 2010). Even three exceptional readers who maintained standardised single word reading scores of over 90 for 2 years, achieving reading ages around 8 years, made no more progress on these measures than limited readers.

Whereas PA and syntactic understanding are relative weaknesses in DS (Lemons & Fuchs, 2010; Chapman et al., 1991), it was possible that the relative strength in receptive vocabulary associated with DS would help readers to elaborate their vocabulary knowledge. As a correlate of CA (Laws et al., 2014), depth of vocabulary grows with real world experience and this could plausibly encompass experience through reading. However, the vocabulary depths of the readers did not differ from those of children whose reading skills remained limited at Time 2.

The present studies showed that reading comprehension in DS depends on word reading and listening comprehension abilities, just as in typical development, but listening comprehension appears to assume more importance at an earlier stage than in typical development. PA was also a factor in reading comprehension in DS, beyond its influence on word reading and sentence recall. Across both studies, listening as well as reading comprehension test results were affected by children’s particular difficulties in understanding more complex grammar and in deriving meanings that depend on inference. Study 2 provided no evidence of reciprocal relationships to suggest that learning to read was the way to improve oral language comprehension. Results suggest rather that intervention should target oral language skills to boost reading comprehension. Since PA predicted reading and reading comprehension, PA appears an appropriate target for training. However, while some children with DS have benefitted from PA interventions, the skills do not always transfer to reading untaught words (Lemons & Fuchs, 2010; Burgoyne et al., 2012). Burgoyne et al. (2012) combined reading and language training, aiming to improve PA by teaching letter knowledge and phoneme blending directly, and indirectly by teaching vocabulary since, theoretically, vocabulary growth has potential to support reading development by shifting lexical representations from whole words to segments of words or to the individual phonemes essential to children’s use of the alphabetic strategy. Despite statistically significant increases in letter knowledge and phoneme blending skills in the intervention group compared to controls, PA skills were not engaged to decode nonwords or, by implication, words (Burgoyne et al., 2012). The intervention did not address reading comprehension but these results, and a poor response to teaching receptive vocabulary despite an intensive and sustained programme of intervention, suggest that targeting oral language skills and PA to boost children’s reading comprehension may not be not straightforward.

The results of the present and previous studies suggest that, to ensure understanding, reading materials for learners with DS should be selected to provide texts appropriate for their oral language level. However, Cologon (2013) has advised that children may become frustrated if restricted to books that they can easily understand. Cologon (2013) reported success with what she termed a holistic approach that focused on supporting the development of decoding skills and linking reading to meaning. The program produced convergence of word identification and reading comprehension abilities after 2 years and these continued in parallel for a further 2.5 years. However, this report was limited to the study of one boy’s reading progress. Given the range of individual differences in children with DS, evident even within the relatively small group included in the present studies, children are likely to differ in terms of their language and literacy teaching needs. Until there is more research on intervention it is not clear to what extent such results could be more generally achieved.

## Footnotes

1 Stroller in the United States.
